# Coral Uptake of Inorganic Phosphorus and Nitrogen Negatively Affected by Simultaneous Changes in Temperature and pH

**DOI:** 10.1371/journal.pone.0025024

**Published:** 2011-09-16

**Authors:** Claire Godinot, Fanny Houlbrèque, Renaud Grover, Christine Ferrier-Pagès

**Affiliations:** 1 Ecophysiology, Centre Scientifique de Monaco, Monaco, Monaco; 2 Marine Environment Laboratories, International Atomic Energy Agency, Monaco, Monaco; 3 Institut de Recherche pour le Développement, Coreus, Nouméa, Nouvelle-Calédonie; California Academy of Sciences, United States of America

## Abstract

The effects of ocean acidification and elevated seawater temperature on coral calcification and photosynthesis have been extensively investigated over the last two decades, whereas they are still unknown on nutrient uptake, despite their importance for coral energetics. We therefore studied the separate and combined impacts of increases in temperature and *p*CO_2_ on phosphate, ammonium, and nitrate uptake rates by the scleractinian coral *S. pistillata*. Three experiments were performed, during 10 days i) at three pH_T_ conditions (8.1, 7.8, and 7.5) and normal temperature (26°C), ii) at three temperature conditions (26°, 29°C, and 33°C) and normal pH_T_ (8.1), and iii) at three pH_T_ conditions (8.1, 7.8, and 7.5) and elevated temperature (33°C). After 10 days of incubation, corals had not bleached, as protein, chlorophyll, and zooxanthellae contents were the same in all treatments. However, photosynthetic rates significantly decreased at 33°C, and were further reduced for the pH_T_ 7.5. The photosynthetic efficiency of PSII was only decreased by elevated temperature. Nutrient uptake rates were not affected by a change in pH alone. Conversely, elevated temperature (33°C) alone induced an increase in phosphate uptake but a severe decrease in nitrate and ammonium uptake rates, even leading to a release of nitrogen into seawater. Combination of high temperature (33°C) and low pH_T_ (7.5) resulted in a significant decrease in phosphate and nitrate uptake rates compared to control corals (26°C, pH_T_ = 8.1). These results indicate that both inorganic nitrogen and phosphorus metabolism may be negatively affected by the cumulative effects of ocean warming and acidification.

## Introduction

Ocean acidification is the result of anthropogenic carbon dioxide (CO_2_) emissions partially dissolving into seawater and progressively declining its pH [1]: over the 20^th^ century, the oceans' average pH_T_ (total scale) has decreased by 0.1 unit from 8.21 to 8.10 [2], [3], and it is predicted to further decrease by 0.3-0.5 unit by the end of this century [2], [4]. The majority of calcifying organisms, and particularly scleractinian corals, are negatively affected by ocean acidification, as shown by the decrease in calcification, which is one of the main processes, with photosynthesis, studied up to now in this context. A drop in pH is indeed known to affect the carbonate cycle [4]–[6], reducing carbonate ions that corals use to build their skeleton, and leading to reduced coral calcification rates [1], [7]–[10].

Beyond its impact on the carbonate cycle, ocean acidification also alters other elemental cycles, such as those of nitrogen and phosphorus [11]–[14]. Both nutrients are however essential for coral metabolism. Indeed, reef-building corals are living in nutrient-poor tropical waters, where the supply of available nutrient sources (zooplankton, dissolved and particulate organic matter, inorganic nutrients) is generally low [15]. Yet, by an efficient nutrient recycling between reef biota, coral reefs retain a high productivity. Corals have for instance adapted to their oligotrophic environment by developing a symbiosis with dinoflagellates of the genus *Symbiodinium*, commonly called zooxanthellae. These symbionts largely contribute to the nutrition of their animal host by providing 1) photosynthesis-derived carbon to the animal tissue [16]; and 2) essential nutrients, such as nitrogen and phosphorus, either directly taken up from the external environment or recycled from the animal wastes [17]. These nutrients are combined to the products of photosynthesis and are transferred back to the host, mainly in the form of essential amino acids for nitrogen [18]. It has been calculated that uptake of inorganic nitrogen, at natural concentrations, contributes approximately 30% to the daily nitrogen requirement of the species *Acropora palmata* for gamete and mucus production, growth, and tissue repair [19]. In another coral species, *Pocillopora damicornis*, uptake of ammonium could even completely satisfy the nitrogen demand of this coral at field concentrations [20]. Concerning phosphorus, it enters into the composition of many biological molecules (DNA, RNA, phospholipids) and has a role in several biochemical mechanisms (through ATP). It controls in part coral growth and zooxanthellae photosynthesis [21], [22]. The issue of nutrient limitation of corals and their symbionts is therefore of prime interest, as nutrient provision sustains corals' metabolism in general, and in particular during thermal bleaching events. It has indeed been shown that nutrient-repleted corals, with higher zooxanthellae density and photosynthetic rates, are less susceptible to bleaching or nutrient shortage [23], [24]. Provision of nutrient to corals is also important for the entire reef, because healthy corals sustain a high reef biomass production.

Despite the importance of nutrient provision for coral physiology and coral reef functioning, and its potential alteration by ocean acidification, only three studies have reported on the impacts of pH on nutrient uptake by symbionts or symbiotic cnidarians, and none of them have considered these effects in the view of ocean acidification. Rahav et al. [25] showed that the activity of glutamate dehydrogenase, an enzyme involved in the assimilation of ammonium, was higher at pH_T_ 7.3 than at pH_T_ 8.1 in the coral *S. pistillata*. D'Elia et al. [26] reported that ammonium uptake was insensitive to a pH_T_ decrease between 8.8 and 7.8 in zooxanthellae freshly isolated from the giant clam *Tridacna crocea*. A final study showed that ammonium accumulated more in the hemolymph of the giant clam *Tridacna gigas* at pH_T_ 7.4 than at pH_T_ 7.9 [27]. Therefore, the first aim of our study was to test if a decrease in external pH changed the uptake rates of inorganic nitrogen and phosphorus by the scleractinian coral *S. pistillata*. The impacts might be due to changes in the coral metabolism with acidification, which would in turn i) modify nutrient requirements as known in phytoplankton [13], [28] and higher plants [29]–[31], or ii) decrease the energy expended for nutrient uptake, as may be the case if more energy is used for calcification under acidified conditions [32], [33]. The impacts may also be related to changes in the relative abundances of the protonated species of nitrogen and phosphorus. Indeed, phosphate is transported via a sodium/phosphate symporter in the coral *Stylophora pistillata* [34], but the ability of this transporter to discriminate among the protonated species is still unknown. For nitrogen, no studies to date have elucidated the transporters involved in ammonium and nitrate uptake in corals. However, the potential impact of speciation changes on nutrient transport cannot be neglected.

Besides oceanic acidification, anthropogenic CO_2_ emissions have also driven an increase in the oceans' average temperature by 0.4–1.0°C in the past four decades [2], [35]. Seawater temperatures are predicted to further rise, and cause several mass coral mortalities over the century [36], [37]. Indeed, as tropical corals usually live close to their upper thermal limit, they are very sensitive to exposure to elevated temperature [36], [38]–[40]. One of the first responses of corals to thermal stress is bleaching, the disruption of the symbiotic association and the expulsion of the zooxanthellae [36], [40]–[43], which in turn affects coral calcification [7], [9], [44], [45] and photosynthesis [9], [41], [46]–[48]. As for acidification, the impacts of elevated seawater temperature on the above two processes have been extensively studied in the last thirty years, but the effects on the nutrient requirements have never been examined. As temperature controls enzymatic activities, ocean warming could potentially impact nutrient uptake in corals. The second aim of this study was therefore to test the effects of a thermal stress on the uptake rates of inorganic nitrogen and phosphorus by the coral *S. pistillata*. Finally, we also tested both effects of oceanic acidification and elevated temperature on these uptake rates, since the two stressors are likely to act simultaneously on reefs in the future [48].

The general aim of the present study was to test, for the first time, the effects of increases in temperature and *p*CO_2_ of magnitudes similar to those expected by 2100 and beyond on the nutrient uptake rates of the scleractinian coral *S. pistillata*. This study thus enriches our existing predictive capabilities on corals' response to future changes.

## Methods

### Organisms and culture conditions

Colonies of the zooxanthellate coral *S. pistillata* were initially obtained from the Red Sea. Methods of collection were reviewed and approved by CITES under the permit number DCI/89/32, and importation was performed under CITES permit number 125/SPV. Colonies were then cultured at the Centre Scientifique de Monaco (CSM), under controlled conditions (26°C, salinity of 38). 1.5 month before the start of the experiment, the ca. 220 nubbins (i.e. branch tips) used during the experiment were prepared by cutting branches (2.5±1.0 cm long and 0.6±0.3 cm in diameter) of about twenty parent colonies with pliers after Tambutté et al. [49]. Nubbins were attached on nylon wires and suspended in aquaria until tissues fully covered the skeleton. They were lightly fed once a week with *Artemia salina* nauplii. In order to avoid the additional and undesired effects of bleaching on the impacts of a pH- and/or a temperature-stress, corals were maintained under a low photosynthetic active radiation (PAR) of 110* µ*mol m^−2^ s^−1^,using metal halid lamps (Philips, HPIT, 400W, Eindhoven, The Nederlands), with a photoperiod of 12h:12h light:dark, and measured using a spherical quantum sensor (LiCor LI-193), with a 12 h:12 h dark:light cycle. Nubbins were then randomly assigned to their experimental 40-L tanks and allowed to acclimate for a week. Temperature was individually regulated in each aquarium at 26.0±0.1°C during the acclimation period, and pH was 8.1. Aquaria were continuously provided with unfiltered seawater, with a flow rate of ca. 40 L day^−1^.

### Experimental design

To examine the effects of exposure to lowered pH and/or elevated temperatures on the uptake rates of ammonium, nitrate, and phosphate by *S. pistillata*, three experiments were performed, in which nubbins were incubated for 10 days i) at three pH_T_ conditions (8.1, 7.8, and 7.5) at normal temperature (26°C), ii) at three temperature conditions (26°, 29°C, and 33°C) at normal pH_T_ (8.1), and iii) at three pH_T_ conditions (8.1, 7.8, and 7.5) at elevated temperature (33°C). A short-term exposure of 10 days was chosen to be able to compare our results with previous ones. Indeed, it is usually the length of a thermal stress above 30°C, either observed *in-situ* [50] or applied in laboratory experiments [23], [51], [52]. A longer thermal stress generally induces a complete bleaching of the corals (and therefore a complete change in their physiology) and might lead to their death. A *p*CO_2_ stress of 10 days also ranges in the mean culture length of previous experiments (mean of 12 days in the comprehensive Table 2 from Erez et al. 2011 if studies lasting over a c.a. year are excluded) [33].

**Table 2:** Effect of pH and/or temperature on coral PSII photosynthetic efficiency.

	Temperature		
**pH_T_**	**26°C**	**29°C**	**33°C**
**7.5**	0.65±0.002		0.62±0.021
**7.8**	0.65±0.004		0.63±0.009
**8.1**	0.66±0.015	0.69±0.002	0.63±0.011

Effect of a 10-day exposure to lowered pH and/or elevated temperatures on the maximum quantum yield of photosystem II (F_v_/F_m_ of PSII) of *Stylophora pistillata* nubbins. Corals were incubated either under 3 different pHs at 26°C, under 3 different temperatures at pH_T_ = 8.1, or under 3 different pH_ T_ at 33°C. Data are presented as mean ± SE of 5 to 10 nubbins per condition.

Corals were not fed during the 10-day incubations, in order to minimize the impact of organic nutrients on uptake rates. To avoid any undesirable “tank” effects, the aquaria were carefully cleaned once a week to minimize algal growth on the walls and nylon wires. Salinity and irradiance were also monitored during the course of the experiments. This maintenance ensured that similar conditions prevailed in all the tanks, except for the fixed parameters (pH and seawater temperature). The three experiments were set up using 3 conditions with duplicated aquaria.

In experiment i), aquaria were maintained at 26±0.2°C and 3 different pHs (a normal pH_T_: 8.09±0.04, i.e. 378* µ*atm CO_2_; a pH_T_ level projected for the end of the century: 7.78±0.06, i.e. 903* µ*atm CO_2_; and a very low pH_T_ level: 7.46± 0.04, i.e. 2039* µ*atm CO_2_). The pH was controlled using a pH-stat system (IKS, Karlsbad, accuracy ± 0.05 pH_T_ unit) by bubbling independently pure CO_2_ in each tank that was continuously aerated with CO_2_-free air. A temperature of 26°C was kept constant inside each aquarium using heaters connected to electronic controllers (Biotherm, ± 0.2°C accuracy) and was logged at 10-min intervals using individual temperature recorders (Seamon).Corals were maintained 10 days under these conditions before their nutrient uptake rates were measured.

In experiment ii), aquaria were maintained at the control pH_T_ (8.1) and at 3 different temperatures (a normal temperature: 26.0±0.2°C, the temperature projected for the end of the century: 29.0±0.2°C; and a very high temperature: 33.0±0.2°C). Temperature, salinity and irradiance were also repeatedly monitored over the course of the experiment, and the maintenance procedure was the same as in experiment i). Corals were also maintained 10 days under these conditions before the nutrient uptake measurements. Before the beginning of the 10-days incubation period, for the two tanks in which temperature was increased to 33°C, corals were first acclimated for 7 days at 29°C then 2 days at 31°C and 2 additional days at 33°C. This gradual temperature increase over 11 days prevented any thermal shock and coral mortality.

Finally, experiment iii) was performed using the same design as in experiment i), except that the temperature was set to 33°C (using the same gradual procedure as in experiment ii) for all the aquaria).

Data of seawater temperature and carbon chemistry were measured as in Houlbrèque et al. [53] and are presented in Table 1. Briefly, three 20 mL samples of seawater were collected daily in each tank, filtered through 0.45 µm GF/F Whatman filters, poisoned with 0.05 ml of 50% HgCl_2_ to avoid biological alteration, and finally stored in glass bottles in the dark at 4°C. pH_T_ was measured using a Metrohm, 826pH meter, equipped with a glass electrode calibrated on the total scale using Tris/HCl and 2-aminopyridine/HCl buffer solutions with a salinity of 38 [54]. Means pH_T_ were calculated from hydrogen ion concentrations of each measurement and then re-converted back to pH [54]. These measurements were used to adjust every day the pH values of the pH-stat system. Total alkalinity (TA) was calculated from the Gran function. Titrations of TA standards were within 0.7 µmol kg^−1^ of the nominal value. Mean TA of seawater was 2508±16 µmol kg^−1^and remained stable during the whole experiment. *p*CO_2_ was calculated from pH_T_, TA, temperature and salinity using the free-access CO_2_ Systat package. For each experimental treatment, the parameters of the carbonate system remained constant over the 10-days exposure periods (repeated measures ANOVA, all *p*>0.05).

**Table 1:** Carbonate system parameters during the experiments.

Experiment	Temperature	pH_T_	pCO_2_	HCO_3_^-^	CO_3_^2-^	CO_2_	DIC
	(°C)		(µatm)	(µmol kg^−1^)	(µmol kg^−1^)	(µmol kg^−1^)	(µmol kg^−1^)
**1) Effect of pH**	26.0±0.2	8.09±0.04	378±15	1890±4	267.7±2.0	55.2±2.0	2213±2.2
	26.0±0.2	7.78±0.06	903±51	2220±48	150.1±4.7	25.1±1.2	2395±16.7
	26.0±0.2	7.46±0.04	2039±82	2345±6	80.6±2.6	11.8±0.2	2437±5.5
**2) Effect of temperature**	26.0±0.2	8.08±0.02	375±21	1885±10	268.7±1.6	54.7 ±2.2	2208±6.0
	29.0±0.2	8.09±0.01	405±17	1891±4	270.2±2.7	59.1±1.8	2220±3.1
	33.0±0.2	8.07±0.04	429±34	1878±8	269.5±1.9	62.6±1.5	2210±4.2
**3) Effect of pH x temperature**	33.0±0.2	8.08±0.01	434±25	1892±5	269.3±2.1	63.3±1.7	2225±3.9
	33.0±0.2	7.76±0.02	1037±42	2210±34	155.4±5.3	28.9±1.5	2394±14.8
	33.0±0.2	7.45±0.01	2242±75	2337±10	82.8±2.9	12.9±0.1	2433±4.5

Temperature and parameters of the carbonate system in each treatment during a 10-day exposure to lowered pH and/or elevated temperatures. The values reported are averages ± SE of n = 60measurementsperformed during the 10-days exposure periods. The total alkalinity was constant and equal to 2508±16 ìmol kg^−1^. *p*CO_2_ was calculated from pH_T_, TA, temperature, and salinity using the free-access CO_2_ Systat package.

### Measurements of nutrient uptake rates

After 10 days of incubation, inorganic nutrient uptake was studied by following the depletion of phosphate, ammonium, or nitrate over time in beakers containing filtered-seawater enriched with the corresponding nutrient. For each nutrient (PO_4_, NH_4_, or NO_3_), six 200-mL beakers were maintained in a water bath at the pH, temperature and irradiance to which the nubbins were previously exposed. Five randomly-selected coral nubbins per experimental condition, each suspended to a nylon wire, were introduced in separate beakers. The last beaker, without coral, served as a control for changes in nutrient concentrations due to adsorption onto beaker surface, to consumption by microbial activity, or to air contamination (particularly for ammonium). After a 10–30 min acclimatization period, beakers were enriched with 300* µ*L of a 10-mmol L^−1^ buffer solution of KH_2_PO_4_, NH_4_Cl, or KNO_3_ to reach an initial concentration of 3* 7*mol L^−1^. This concentration was chosen because it corresponded to the plateau in previous concentration-dependent uptake experiments with the same coral species [34], [55]. Magnetic stirring bars ensured proper homogenization of nutrient inside the beakers. A 10-mL water sample was taken from each beaker immediately after enrichment and then every 15 min for 60 min. 10-mL samples were also taken in the beakers at the beginning and at the end of the incubation in order to verify that pH had not changed during that period. pH values were measured on total scale (pH_T_) with a glass electrode connected to a pH meter (Metrohm, 826 pH mobile) calibrated on the total scale using Tris/HCl and 2-aminopyridine/HCl buffers with a salinity of 38 [54].

Ammonium and phosphate levels were determined manually, according to the spectrofluorimetric method of Holmes et al. [56], and using the ascorbic acid technique of Murphy and Riley [57], respectively. Levels of nitrate were determined using an autoanalyzer (Axflow) according to Aminot and Kérouel [58]. Uptake rates of ammonium, nitrate, and phosphate were calculated as the quantity taken up by each nubbin in 60 min, after correction of concentrations for the diminution of the beakers' volume and after having verified that the uptake rates were linear during the first hour. Uptake rates were normalized to the total chlorophyll content, to the zooxanthellae concentration, or to the surface area of the nubbins (see below).

### Measurements of physiological parameters

Photosynthesis and respiration were measured at the end of the experiments, for 3 randomly selected corals per experimental condition, using the same method as described in Godinot et al. [55]. Seawater in the respirometric chambers was at the same pH and temperature as the corresponding experimental tanks. Photosynthesis was measured at a PAR of 110 and 300* µ*mol m^−2^ s^−1^. Data were normalized to the surface area of the nubbins and to their chlorophyll content (see below). Additionally, we measured the maximum quantum yield of photosystem II (F_v_/F_m_ of PSII) and the electron transport rate of PSII (ETR = dark adapted F_v_/F_m_ × 0.5 × PAR) at 9 different light intensities (0 – 3000* µ*mol m^−2^ s^−1^ PAR) at the end of the two experiments, for 5 randomly selected corals per experimental condition, using pulse-amplitude-modulated chlorophyll fluorometry (diving PAM, Waltz, Germany).

Chlorophyll (chl), protein (prot), and zooxanthellae (zoox) concentrations were measured at the end of the experiments, on all nubbins used for the uptake rate measurements (15 nubbins per experimental condition). Tissues and zooxanthellae were striped from the skeleton in 10 mL of filtered seawater using an Air-Pik, then homogenized using a Potter grinder, and divided into 3 aliquots for chlorophyll, zooxanthellae, and protein measurements. Chlorophyll *a* and *c2* were extracted in 99% acetone (24 h at 4°C). The extracts were centrifuged at 11,000 x g for 10 min at 4°C, and the absorbances measured at 630, 663, and 750 nm. Concentrations were computed according to the 100% acetone equations of Jeffrey and Humphrey [59]. Zooxanthellae were counted using a counting chamber and an improved version of the Histolab v.5.2.3 image analysis software (Microvision, Every, France). Proteins were extracted in 0.5 mol L^−1^ NaOH (60°C, 5 h) and measured using the commercially available BC Assay Interchim kit, and results analyzed with the GENESIS software (v.3.3). Results were normalized to the surface area of nubbins, measured with the wax technique [60].

### Statistical analyses

Equality of variances and normality of residuals were tested using Levene and Shapiro-Wilk tests (Statgraphics Centurion version 15). Data from the tanks replicated between the 3 experiments (26°C or 33°C, and pH_T_ of 8.1) were compared using unpaired *t*-tests. Since there was no significant difference between replicated tanks, results from those tanks were pooled together for the subsequent statistical analyses. Effects of temperature and pH on nutrient uptake rates, areal chlorophyll, zooxanthellae and protein content, photosynthesis, respiration, F_v_/F_m_, and ETR_max_were examined using analyses of variance tests (2-ways ANOVAs, with temperature and pH as fixed factors; StatView version 5.0). When significant differences were found, ANOVAs were followed by protected least significant difference (PLSD) Fisher post hoc tests to attribute differences between specific treatments. The effect of temperature alone on the above-cited parameters was tested using a 1-way ANOVA.

## Results

### Effect of lowered pH or/and elevated temperature on physiological parameters

**Figure 1:**
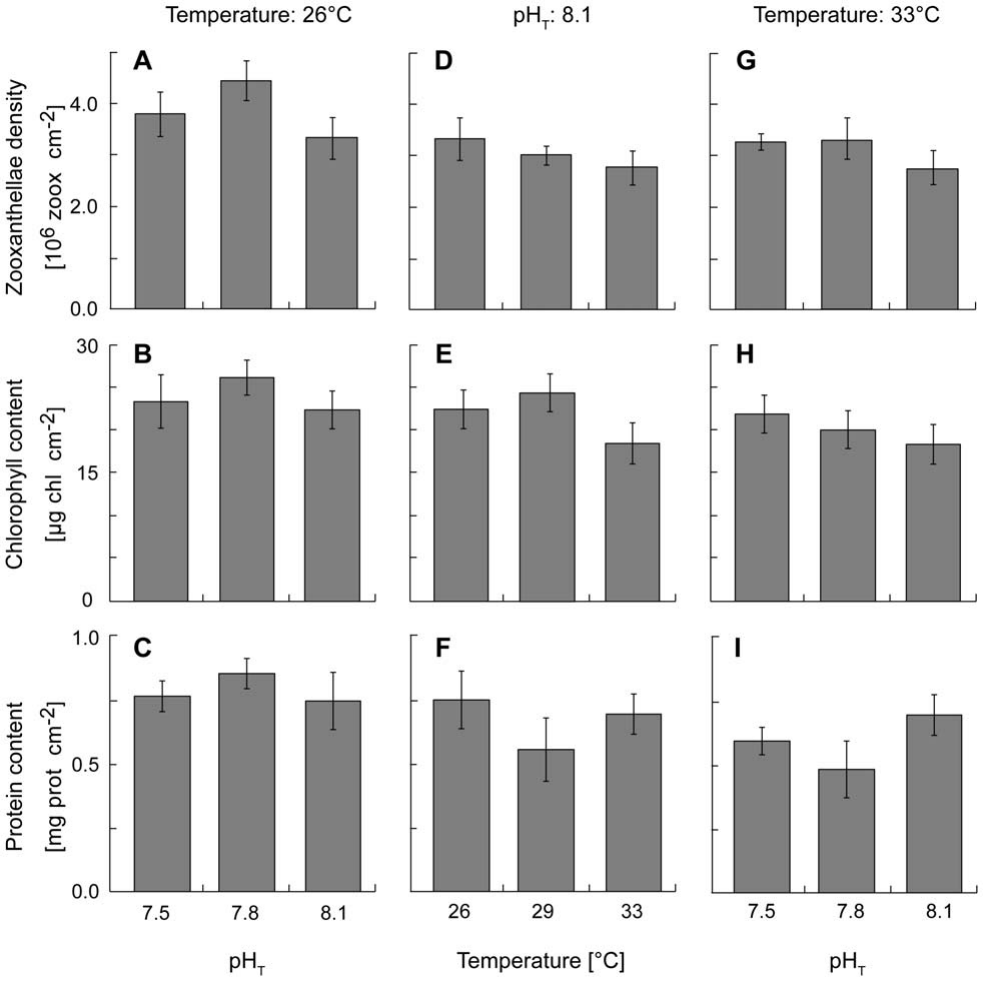
Effect of pH and/or temperature on coral biomass. Effect of a 10-day exposure to lowered pH and/or elevated temperatures on zooxanthellae (**A**, **D**, **G**), chlorophyll (**B**, **E**, **H**), and protein (**C**, **F**, **I**) content of *Stylophora pistillata* nubbins. Corals were incubated either under 3 different pH_T_ at 26°C (**A**, **B**, **C**), under 3 different temperatures at pH_T_ = 8.1 (**D**, **E**, **F**), or under 3 different pH_T_ at 33°C (**G**, **H**, **I**). Data are presented as mean ± SE of 15 nubbins per treatment.

**Figure 2:**
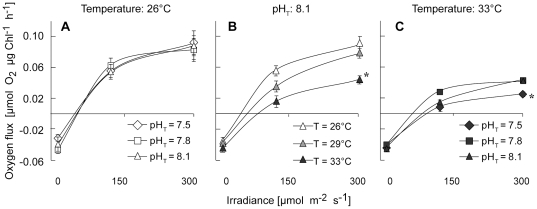
Effect of pH and/or temperature on coral photosynthesis and respiration. Effect of a 10-day exposure to lowered pH and/or elevated temperatures on oxygen fluxes in *Stylophora pistillata* nubbins. Corals were incubated either under 3 different pH_T_ at 26°C (**A**), under 3 different temperatures at pH_T_ = 8.1 (**B**), or under 3 different pH_T_ at 33°C (**C**). Data are presented as mean ± SE of 3 nubbins per treatment. Lozenges: pH_T_ = 7.5, squares: pH_T_ = 7.8, triangles: pH_T_ = 8.1, open symbols: T = 26°C, gray symbols: T = 29°C, dark symbols: T = 33°C. Stars indicate treatments significantly different from the control (pH_T_ = 8.1 for **A** and **C**; T = 26°C for **B**).

**Figure 3:**
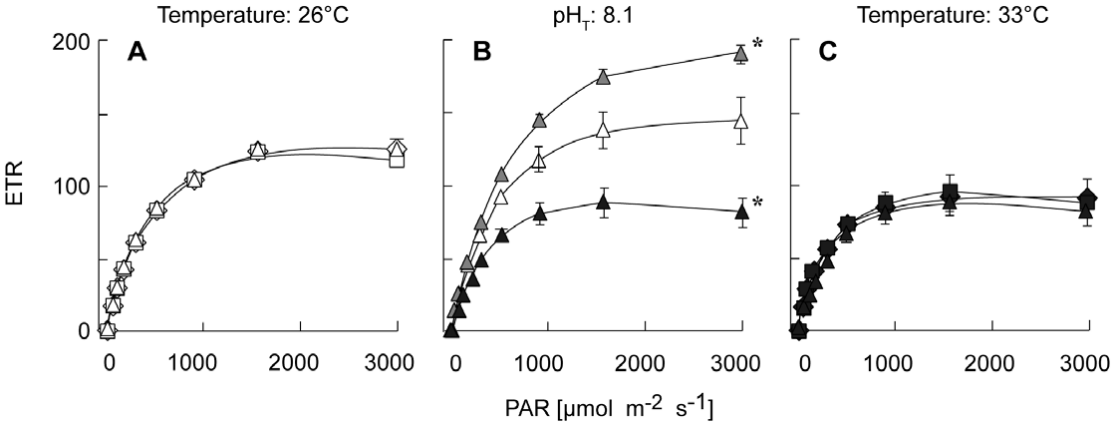
Effect of pH and/or temperature on coral PSII photosynthetic activity. Effect of a 10-day exposure to lowered pH and/or elevated temperatures on the electron transport rate (ETR) of *Stylophora pistillata* nubbins. Corals were incubated either under 3 different pH_T_ at 26°C (**A**), under 3 different temperatures at pH_T_ = 8.1 (**B**), or under 3 different pH_T_ at 33°C (**C**). Data are presented as mean ± SE of 5 nubbins per treatment. Symbols are the same as on Fig. 2. Stars indicate treatments significantly different from the control (pH_T_ = 8.1 for **A** and **C**; T = 26°C for **B**).

After 10 days of incubation, there was no effect of either pH, temperature, or both on the areal content of zooxanthellae (Fig. 1a,d,g), chlorophyll (Fig. 1b,e,h), and protein (Fig. 1c,f,i), nor on the rates of respiration normalized to the chlorophyll content (Fig. 2; 1-way and 2-ways ANOVA, all *p*>0.05). The rate of photosynthesis normalized to the chlorophyll content was not affected by a change in pH at the control temperature of 26°C (Fig. 2a), but was impacted by a change in temperature alone (Fig. 2b) or combined with a low pH (Fig. 2c). Therefore, at pH_T_ = 8.1, photosynthesis was ca. 2 times lower at 33°C than at 26°C or 29°C (2-ways ANOVA, *p* = 0.0004, *F*_2_ = 17.51). At 33°C, photosynthesis was also 1.8 times lower for pH_T_ = 7.5 than for pH_T_ = 7.8 and 8.1 (2-ways ANOVA, *p* = 0.038, *F*_2_ = 4.48). There was however no significant interaction of pH and temperature on these rates of photosynthesis (2-ways ANOVA, *p* = 0.28, *F*_2_ = 1.37 for an irradiance of 300* µ*mol m^−2^ s^−1^). Results were the same when respiration and photosynthetic rates were normalized to the skeletal surface area (cm^2^). There was no effect of pH on F_v_/F_m_ and ETR, both at 26°C and 33°C (Table 2 for F_v_/F_m_, and Fig. 3a,c for ETR; 2-way ANOVAs, *p* = 0.93, *F*_2_ = 0.07 for F_v_/F_m_; and *p* = 0.87, *F*_2_ = 0.14 for ETR). Conversely, temperature had an effect at pH_T_ = 8.1 (Table 2 for F_v_/F_m_, and Fig. 3b for ETR; 1-way ANOVAs, *p* = 0.01, *F*_2_ = 7.52 for F_v_/F_m_; *p*<0.0001, *F*_2_ = 24.70 for ETR): F_v_/F_m_ and ETR were significantly higher at 29°C than at 26°C (PLSD Fisher tests, both *p*<0.02, df = 9), and they were significantly lower at 33°C than at 26°C (PLSD Fisher tests, both *p*<0.001, df = 9). There was however no significant interaction of pH and temperature on these parameters (2-ways ANOVAs, *p* = 0.67, *F*_2_ = 0.40 for F_v_/F_m_; *p* = 0.51, *F*_2_ = 0.69 for ETR).

### Effect of lowered pH or/and elevated temperature on nutrient uptake rates

Results of the statistical tests were the same regardless of the normalization used for uptake rates. Uptake data in this paper are presented as normalized to the chl content.

**Figure 4:**
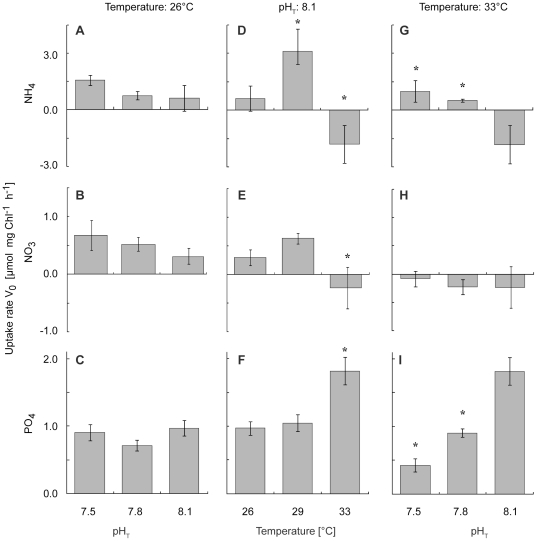
Effect of pH and/or temperature on coral nutrient uptake rates. Effect of a 10-day exposure to lowered pH and/or elevated temperatures on ammonium (**A**, **D**, **G**), nitrate(**B**, **E**, **H**), and phosphate (**C**, **F**, **I**) uptake rate of *S. pistillata* nubbins. Corals were incubated either under 3 different pH_T_ at 26°C (**A**, **B**, **C**), under 3 different temperatures at pH_T_ = 8.1 (**D**, **E**, **F**), or under 3 different pH_T_ at 33°C (**G**, **H**, **I**). Data are presented as mean ± SE of 5 nubbins per treatment. Stars indicate treatments significantly different from the control (pH_T_ = 8.1 for **A**, **B**, **C**, **G**, **H**, **I**; T = 26°C for **D**, **E**, **F**).

A decrease in pH at 26°C (Fig. 4a,b,c) did not significantly affect ammonium (2-ways ANOVA, *p* = 0.29, *F*_2_ = 1.35), nitrate (2-ways ANOVA, *p = *0.39, *F*_2_ = 1.03), and phosphate (2-ways ANOVA, *p = *0.11, *F*_2_ = 2.49) uptake rates.

Conversely, elevated temperatures had a significant effect on nutrient uptake. Indeed, under a normal pH_T_ of 8.1 (Fig. 4d,e,f), ammonium uptake rates first increased by 5 fold at 29°C compared to 26°C (PLSD test, *p* = 0.004), but then severely decreased to negative values (i.e. release of inorganic nitrogen) at 33°C (1-way ANOVA, *p = *0.01, *F*_2_ = 6.39). An equivalent decrease to negative values at 33°C was also observed for nitrate (1-way ANOVA, *p = *0.05, *F*_2_ = 3.63), while phosphate uptake rates almost doubled at 33°C (1-way ANOVA, *p = *0.003, *F*_2_ = 9.62).

At 33°C and a low pH_T_ of 7.5 (Fig. 4g,h,i), ammonium uptake rates were the same as at pH_T_ of 8.1 and temperature of 26°C (Fig. 4a,d), and there was no significant interaction of pH and temperature (2-ways ANOVA, *p = *0.10, *F*_2_ = 2.56). On the contrary, corals excreted nitrate at 33°C and a low pH_T_ of 7.5, and release rates did not vary with pH (2-ways ANOVA, *p = *0.88, *F*_2_ = 0.14). Conversely, phosphate uptake rates severely decreased at 33°C and a low pH_T_ of 7.5, by 4 fold when compared to 33°C and a normal pH_T_ of 8.1 (2-ways ANOVA, *p*<0.0001, *F*_2_ = 27.21), or by 2 fold when compared to control corals (pH_T = _8.1, 26°C; 2-ways ANOVA, *p*<0.0001, *F*_2_ = 22.78). There was a significant interaction of pH and temperature (2-ways ANOVA, *p*<0.0001, *F*_2_ = 20.56) on phosphate uptake rates.

## Discussion

This study presents the changes in the uptake of inorganic phosphorus and nitrogen by a scleractinian coral, *S. pistillata,* under elevated temperature and/or decreased pH, thus bringing new insights into the ability of corals to respond to changes in their environment. To the best of our knowledge, the effects of acidification and/or elevated temperature on nutrient uptake by cnidarians, or even phytoplankton species, have been poorly investigated up to date. We showed that a short-term seawater acidification alone had no impact on the nutrient uptake rates, while elevated temperature (33°C) increased the uptake of phosphate but severely decreased, and even reversed the uptake of nitrogen (nitrogen release). An elevation in both temperature and *p*CO_2_ significantly decreased the uptake of phosphate and nitrate compared to normal conditions (pH_T_ 8.1 and 26°C), suggesting that overall, climate change will negatively affect nutrient supply in corals.

### Effect of lowered pH or/and elevated temperature on physiological parameters

Even though corals did not bleach during the 10-days incubation under high temperature, photosynthesis was impacted, with an increase in the F_v_/F_m_ and ETR at 29°C, and a decrease of the photosynthetic rates, F_v_/F_m_, and ETR at 33°C, as previously observed [61]. A general decrease in photosynthesis has been observed when temperatures extend above 31°C [41], [46], [47], [62], [63], whereas a positive effect was reported when temperatures remained below 31°C [9], [41], [46], [48], [62], [64]. The decrease in the rates of photosynthesis is often due to damages to the photosystems II (PSII) early during temperature stress [41], [46], [62], [65], [66], as observed in this study. Conversely to the temperature effect, there was no effect of pH alone on the photosynthetic rates, which is also in agreement with most of the previous studies [8], [9], [53], [67]–[69]. This feature is attributed to the fact that corals do not rely solely on dissolved CO_2_ for photosynthesis, but also largely depend on HCO_3_^-^ [67], [70]–[73]. Our results are however in disagreement with the study of Anthony et al.[48], who reported a decrease in the net productivity and an increase in the respiration of the corals *Acropora intermedia* and *Porites lobata* with seawater acidification even at normal temperature. However, the latter study used a higher natural irradiance (1000* µ*mol photons m^−2^ s^−1^) and a long-term exposure (8 weeks), which probably brought corals closer to their bleaching threshold. Finally, Hii et al. [74] reported that seawater acidification decreased photosynthesis in the coral *Galaxea fascicularis* and had no impact on that of *Porites cylindrica*, thus showing that the response of corals may be species-dependent.

### Effect of lowered pH or/and elevated temperature on nutrient uptake rates

Maximum nutrient uptake rates correspond, as for all enzyme-involving processes, to an optimum in temperature and pH conditions [75], [76]. This optimum can be different according to the nutrient or the organism considered. In this study, the optimum uptake rate for ammonium was achieved for the normal pH_T_ of 8.1 and a temperature of 29°C, while the highest phosphate uptake rate was observed for the normal pH_T_ of 8.1 and a temperature of 33°C. An increase in nitrate uptake rates was also observed at 29°C, although it was not significant. For both inorganic nitrogen and phosphorus, maximal rates were therefore achieved under elevated temperatures and at the current pH_T_ of 8.1.

#### Effect on ammonium uptake rates

The lack of impact of seawater pH alone (in the range studied) on ammonium uptake rates is in agreement with results reported on zooxanthellae freshly isolated from the symbiotic clam *Tridacna crocea*, for which no difference was found across a pH_T_ range of 7.8 to 8.8 [26]. On the contrary, Fitt et al. [27] observed an inverse relationship between pH and ammonium accumulation in the hemolymph of the symbiotic clam *T. gigas*, over a pH_T_ range of 7.4 to 7.9. However, in the latter study, changes in pH did not occur in the surrounding seawater but directly inside the tissue, and were related to diel variations in zooxanthellae photosynthesis. In our study, ammonium uptake rates displayed a bell-shaped response to temperature-stress, with a 5-fold increase at 29°C and a net decrease, and even release, at 33°C. Since ammonium uptake is a light-stimulated process in symbiotic cnidarians [17], [77]–[80] and is linked to zooxanthellae photosynthesis, the observed increase in uptake rates between 26°C and 29°C and the decrease between 29 and 33°C might be linked to the parallel increase and decrease in photosynthesis and photosynthetic efficiency. A similar release of ammonium was indeed observed in a previous study on the same coral species [25], when the photosynthetic chain was blocked by the electron transport inhibitor DCMU. In that latter study, excretion of ammonium was also elicited by the inhibition of the glutamate synthase (GOGAT) [25], which could be an additional explanation for the observed excretion of NH_4_^+^ at high temperature (33°C). Indeed, although the temperature-sensitivity of this enzyme is not known in corals, heat stress was shown to decrease its activity, as well as that of glutamine synthetase (GS), in higher plants [81]. The release of ammonium at 33°C was however no longer observed under low pH (pH_T = _7.5), and the uptake rate measured under these conditions was comparable to the “control conditions” (pH_T = _8.1, 26°C), therefore suggesting that CO_2_ addition may completely offset the negative impact of temperature. One hypothesis to explain this result is that ammonium-assimilating enzymes (GS, GOGAT, and glutamate dehydrogenase GDH) [25], [80], [82]-[84] in corals are stimulated by seawater acidification, possibly through the impact of acidification on the intracellular pH [85]. For example, the activity of GS, GOGAT, and GDH are influenced by pH in an actinobacteria [86] and a cyanobacteria [87], with pH_T_ optima of 7.0 (GS), 7.2-7.6 (GOGAT), and 7.2-7.5 (GDH). In the coral *S. pistillata*, GDH aminating activity is also pH-dependent, as it increased when pH_T_ was decreased between 8.1 and 7.3 [25]. However, such potential pH effects on the enzymatic activity were only significant at elevated temperature, although a positive but not significant trend was observed at 26°C. It is possible that the enzymatic activity was already optimal at 26°C, as suggested by the concordance of uptake rates measured in this study with those reported in the literature [17], [26], [34], [79], [88], and so acidification did not lead to a significant improvement of ammonium uptake at that temperature. On the contrary, when conditions were no longer optimal (i.e. thermal stress), acidification appears to have a significant alleviating effect. It is therefore possible that, as was demonstrated for calcification [9], the effects of high CO_2_ do not manifest at low temperatures.

#### Effect on nitrate uptake rates

Concerning nitrate, the lack of impact of seawater acidification alone is similar to the response reported for freshwater phytoplankton [89], for pH_T_s ranging between 6.6 and 9.2. Conversely, in some marine macroalgae, some authors [90]–[92] observed an enhancement of nitrate uptake at high CO_2_ levels due to an increase in the activity of the nitrate reductase. Such enhancement may not have occurred in the coral *S. pistillata*, possibly because nitrate is not the main source of nitrogen [93], [94] and therefore, the nitrate reductase might not be very active. As for ammonium, nitrate uptake rates were severely affected by a thermal stress at 33°C, therefore suggesting that the two nitrogen forms present the same temperature optimum for their uptake (i.e. 29°C), and that climate change in general may negatively impact the absorption of nitrogen by scleractinian corals. When seawater acidification was combined to elevated temperature, nitrate uptake rates did not decrease any further.

#### Effect on phosphate uptake rates

The response of phosphate uptake to changes in temperature and pH differed from the nitrogen response. While a thermal stress at 33°C decreased the uptake rates of nitrogen, it increased those of phosphate. The only previous study that examined the impact of seawater temperature on phosphate uptake by the tropical coral *Pocillopora capitata* used a low temperature range of 6 to 22°C [21], and showed that temperatures below 19°C decreased uptake rates. Enhancement of uptake rates by temperature suggests that, although phosphate uptake is light-stimulated [21], [55], [95], [96], it may be less dependent on photosynthesis than ammonium uptake.

As for nitrogen, there was no pH effect alone on phosphate uptake rates. This lack of pH effect (in the same pH range), either on phosphate uptake rates or on the activity of the alkaline phosphatase, has previously been reported for marine phytoplankton [97] and macroalgae [90]. In corals, it is possible that, as for ammonium, the enzymatic activity involved in phosphate uptake and assimilation was optimal at 26°C regarding the metabolic condition of the corals, since the uptake rates were in agreement with rates reported in the literature [21], [34], [55]. When seawater acidification was combined to elevated temperature, uptake rates severely decreased, by 4 fold when compared to elevated temperature and normal pH, or by 2 fold when compared to control corals (pH_T = _8.1, 26°C).These results therefore suggest that a combined thermal and acidification stress might impact the retrieval of phosphate by corals in the future. The temperature-dependent response of corals to acidification may be due to a higher/lower affinity of the carrier for the different phosphate species available under certain environmental conditions. At 33°C and pH_T_ 7.5, the decrease in uptake rates may for instance have resulted from a higher affinity of the carrier towards the phosphate form less available due to more acidic conditions, i.e. the less abundant PO_4_^3-^. Indeed, as seawater pH_T_ decreases from 8.1 to 7.5, the relative abundance of HPO_4_^2-^ increases from 82% to 92%, while PO_4_^3-^ decreases from 18% to 5% (H_2_PO_4_^-^ remains negligible).This 3.6-fold decrease in the relative abundance of PO_4_^3-^ corresponds to the observed 4-fold decrease in phosphate uptake rates. From previous work on *S. pistillata*, we showed that a sodium/phosphate symporter is involved in the uptake of phosphate in this coral [34]. Although the same type of symporter has been reported in a marine aplysia [98], no studies to date have examined the stoichiometry of such transporters in invertebrates. On the contrary, in vertebrates, three distinct families have been found, with a remarkable preference (i.e. higher affinity) for divalent HPO_4_^2-^ in the type II family and for monovalent H_2_PO^4-^ in the type III family [99], [100]. The possibility of preferences for specific phosphate forms therefore needs to be considered more extensively for the sodium/phosphate symporter of corals.

### Conclusion

In conclusion, this study has shown a strong negative impact of increasing temperature on the ability of corals to take up inorganic nitrogen from the surrounding environment. High temperature indeed completely inhibited the uptake of either ammonium or nitrate, and even induced a nitrogen release from the corals. Although high temperature enhanced the uptake of inorganic phosphorus, corals cannot use this phosphorus without nitrogen, which is needed in all metabolic functions, such as tissue growth and repair, chlorophyll synthesis, zooxanthellae growth, reproduction, etc…Although nitrogen can also be acquired through particulate feeding when not available in its inorganic forms, not all coral species are able to efficiently graze on zooplankton [101] or increase their grazing rates when they are under thermal stress [23]. Such species will therefore be the first to suffer from thermal stress. A short-term seawater acidification alone does not seem to induce strong changes in the capacity of corals to take up nutrients; however, longer-term experiments are still needed to confirm these first results and the lack of effect. Nevertheless, when combined to high temperatures, a decrease in seawater pH had in turn a negative impact on phosphate uptake. Since phosphorus is needed in several steps of the photosynthetic machinery [102], [103] as well as in energy production (ATP for example), this study adds to the growing body of evidence that corals will suffer from global change.
